# Association Between the Prognostic Nutritional Index and Outcomes in Patients Undergoing Emergency Laparotomy

**DOI:** 10.3390/jcm15010164

**Published:** 2025-12-25

**Authors:** Sithdharthan Ravikumar, Kasun Wanigasooriya, Shashikanth Vijayaraghavalu, Lanoayo Agbabiaka, Shuker Yahia, Christian Katz, Balasubramanian Piramanayagam, Aravindan Narayanan, Altaf Haji, Muhammad Imran Aslam, Kalimuthu Marimuthu

**Affiliations:** 1 George Eliot Hospital NHS Trust, College Street, Nuneaton CV10 7DJ, UK; kasun.wanigasooriya@geh.nhs.uk (K.W.); shashikanth.vijayaraghavalu@geh.nhs.uk (S.V.); oluwalanoayo.agbabiaka@nhs.net (L.A.); shuker.yahia@geh.nhs.uk (S.Y.); christian.katz@geh.nhs.uk (C.K.); bala.piramanayagam@geh.nhs.uk (B.P.); aravindan.narayanan@geh.nhs.uk (A.N.); altaf.haji@geh.nhs.uk (A.H.); muhammadimran.aslam@geh.nhs.uk (M.I.A.); kalimuthu.marimuthu@geh.nhs.uk (K.M.); 2 Institute of Biomedical Research, College of Medical and Dental Science, University of Birmingham, Vincent Drive, Edgbaston, Birmingham B15 2TT, UK

**Keywords:** nutrition, emergency laparotomy, prognostic nutritional index, surgical outcomes

## Abstract

**Background:** Nutritional status is a key determinant of surgical outcomes, but its assessment in emergency settings remains challenging. The prognostic nutritional index (PNI), which is derived from the serum ALB concentration and lymphocyte count, is a rapid, objective measure of nutritional and immune status. This study evaluated the associations between the PNI and postoperative outcomes in patients undergoing emergency laparotomy. **Methods**: A retrospective observational study was conducted at a single district general hospital in England, including adult patients who underwent emergency laparotomy between January 2019 and December 2023. The PNI was calculated as PNI = serum albumin (g/L) + 0.005 × total lymphocyte count (cells/μL). Patients were classified as malnourished (PNI < 50) or not malnourished (PNI ≥ 50). The outcomes assessed included postoperative complications, length of hospital stay (LOS), 30-day readmission, and three-year all-cause mortality. Statistical analyses included chi-square, Mann–Whitney U, logistic regression, and Kaplan–Meier survival analyses. Preoperative albumin and lymphocyte counts were obtained on admission or within 24 h prior to surgery to calculate the PNI. **Results**: Among 482 patients (median age 68 years; 57% male), 66% were malnourished. Malnutrition was significantly associated with higher ASA grade (*p* < 0.001), frailty (*p* = 0.028), and comorbidity burden (*p* < 0.001). Malnourished patients had longer LOSs (≥12 days; *p* < 0.001) and higher 30-day readmissions (*p* = 0.026). After adjustment for key confounders, low PNI remained independently associated with stoma formation and prolonged length of stay. After adjustment for ASA grade, frailty, comorbidity burden, hypotension, and sepsis, low PNI remained independently associated with stoma formation and prolonged length of stay. Kaplan–Meier analysis revealed reduced three-year survival in malnourished patients (log-rank *p* < 0.01). **Conclusions**: Malnutrition, as defined by a low PNI, is highly prevalent and associated with adverse postoperative outcomes in emergency laparotomy. PNI is a simple, objective, and clinically useful tool that should be incorporated into preoperative assessments to guide early nutritional optimization. However, albumin and lymphocyte counts may be influenced by acute inflammation and resuscitation in emergency presentations, and nutritional interventions were not captured in this retrospective dataset.

## 1. Introduction

Nutritional status is a determinant of surgical outcomes, particularly in patients undergoing major abdominal procedures such as laparotomies [1]. The prognostic nutritional index (PNI), a simple yet effective tool calculated using serum albumin levels and total lymphocyte count, has emerged as a valuable biomarker for assessing preoperative nutritional status and predicting postoperative morbidity and mortality [2,3]. While its prognostic significance has been well-established in elective surgical settings [4], its utility in the context of emergency laparotomies remains underexplored and less well-defined.

Nutrition plays a fundamental role in determining surgical outcomes across both elective and emergency settings. Malnutrition, reported in up to 30–50% of hospitalized surgical patients, is independently associated with impaired wound healing, increased infectious complications, prolonged hospitalization, and increased mortality [5,6,7]. In elective surgery, patients benefit from structured preoperative optimization, including nutritional interventions that have been shown to improve immune competence, reduce complications, and shorten recovery [8]. In contrast, emergency surgical patients often present acutely ill patients, with systemic inflammation or sepsis, limited physiological reserves, and no opportunity for nutritional optimization, rendering even mild nutritional deficits clinically significant [9,10]. Research has consistently shown that poor nutritional status, regardless of the surgical setting, contributes to adverse outcomes and increased postoperative morbidity [11].

Several tools have been developed to assess nutritional risk in surgical patients, including the Body Mass Index (BMI), the Malnutrition Universal Screening Tool (MUST), the Nutritional Risk Index (NRI), the Geriatric Nutritional Risk Index (GNRI), and the Controlling Nutritional Status (CONUT) score [1,2,3,4,5,6,8,9,10,11,12,13,14,15]. While useful in elective care, many of these require subjective inputs, weight history, or complex biochemical data that are often unavailable or unreliable in acute emergencies. BMI, for example, fails to capture muscle wasting or sarcopenic obesity—BMI does not distinguish between lean and fat mass and may overlook sarcopenic obesity, where muscle depletion occurs despite normal or high BMI [16]. Moreover, the MUST and NRI rely on prior weight changes or dietary intake assessments that may not be feasible in urgent settings [17,18]. The CONUT and GNRI scores incorporate serum albumin and lymphocyte count but also depend on cholesterol or anthropometric data, limiting their practicality in time-sensitive emergency environments [19,20].

The prognostic nutritional index (PNI) was therefore chosen for this study because it provides a simple, objective, and reproducible measure based solely on two routinely available laboratory parameters—the serum ALB concentration and total lymphocyte count. PNI integrates both nutritional reserve and immune competence, offering insight into the interplay between metabolic and inflammatory responses to surgical stress [2,3,19,21]. It has been validated across diverse surgical populations, including gastrointestinal, hepatobiliary, and oncologic surgeries, and consistently correlates with postoperative morbidity, infectious complications, and long-term survival [4,6,8]. Compared with other indices, the PNI is simpler to calculate and may be more feasible in emergency settings because it relies only on routinely available admission blood tests; however, this study did not perform a head-to-head comparison of PNI with other nutritional screening tools.

Emergency laparotomy represents one of the most high-risk and resource-intensive emergency general surgical procedures and is often performed in patients with acute intra-abdominal sepsis, bowel obstruction, or perforation. Mortality rates following emergency laparotomy remain substantial, typically ranging from 10 to 30%, with morbidity rates exceeding 40% in some cohorts [9,10,22]. These poor outcomes are attributed to the combination of physiological derangement, advanced age, sepsis, and preexisting comorbidities at presentation [23]. Despite advances in perioperative care and the establishment of national quality improvement initiatives such as the National Emergency Laparotomy Audit (NELA) in the UK, outcome variability persists [11,12]. Nutritional status, often overlooked in emergency settings, may be an important yet modifiable determinant of prognosis in this high-risk population.

In practice, improving recognition of nutritional risk in emergency laparotomy requires rapid, objective screening at the point of admission. Because serum albumin and lymphocyte count are routinely available in emergency blood panels, the PNI can be calculated without additional assessment burden and used to trigger early dietetic review and nutritional support (e.g., early enteral feeding when feasible, or timely parenteral support when enteral nutrition is contraindicated). Early identification and intervention may mitigate postoperative infectious complications, reduce length of stay, and support functional recovery, thereby translating nutritional risk stratification into actionable perioperative care pathways and resource planning (e.g., higher-dependency monitoring).

This study aimed to evaluate the prognostic value of the PNI in patients undergoing emergency laparotomy by analyzing its associations with postoperative complications, length of hospital stay, and overall clinical outcomes. By identifying differences in nutritional risk profiles and surgical outcomes between these two groups, we sought to determine whether the PNI should be routinely incorporated into preoperative assessment protocols to enhance surgical planning and optimize patient recovery.

## 2. Methods

### 2.1. Study Design and Setting

This was a retrospective observational study conducted at a single district general hospital in the UK. The hospital provides an unselected emergency general surgery service supported by a 24 h emergency department and on-site level 2/3 critical care. The study analyzed patient data over a five-year period, from 1 January 2019 to 31 December 2023, during which 482 eligible emergency laparotomy cases were included (approximately 96 cases per year). This study focused on emergency laparotomies performed for upper and lower gastrointestinal tract pathologies.

### 2.2. Ethical Approval and Consent

This audit was registered with the Clinical Governance Team at the George Eliot NHS Trust, UK (ID: 1343). In accordance with national guidance, formal Research Ethics Committee approval was not required for this retrospective observational audit. This was confirmed using the UK Health Research Authority’s “*Is my study research?*” online decision tool (http://www.hra-decisiontools.org.uk/research) (accessed on 16 november 2025).

The study was conducted in accordance with the principles of the Declaration of Helsinki. All patient data were anonymized prior to analysis to ensure confidentiality. Given the retrospective nature of the study, individual patient consent was not required, and institutional approval was obtained in line with local governance protocols.

### 2.3. Study Participation

A total of 623 emergency laparotomy cases were screened during the study period. After exclusions, 482 patients formed the final analytic cohort. The study included patients aged 18 years and over who underwent emergency laparotomy (open or laparoscopic converted to open). Patients who underwent laparoscopic procedures without conversion to open surgery were excluded. “Incomplete records” were defined a priori as cases where preoperative serum albumin and/or lymphocyte count were unavailable within admission laboratory results (preventing calculation of the PNI), and/or where key outcome fields required for analysis (e.g., discharge date required for length of stay) were missing.

### 2.4. Data Collection

The prospectively maintained local copy of the NELA database was queried to identify the patient sample and relevant data. Demographic data, such as age and sex, and preoperative baseline patient characteristic data, such as American Society of Anaesthesiologist (ASA) grade, clinical frailty score and comorbidities, were collated. Sex was recorded as male or female as documented in the NELA database; non-binary gender identity was not captured in the dataset available for this audit. Frailty was recorded using the Clinical Frailty Scale (CFS; Rockwood, 1–9) as documented in the NELA database and was analyzed as <5 versus ≥5. Preoperative NELA mortality scores, hemodynamic parameters (tachycardia and hypotension) and the presence of sepsis were also recorded. Intraoperative details, such as the name of the procedure and stoma formation, were obtained. Data on postoperative outcomes were also obtained. The date of discharge and date of admission were used to calculate the length of stay. Inpatient complications were categorized on the basis of the Clavien–Dindo system and summarized as the presence or absence of complications. Thirty-day readmissions (as captured within our institution’s electronic record/NELA extract) were identified. Planned versus unplanned readmission could not be reliably distinguished within the retrospective dataset, and readmission capture may be limited to episodes recorded at the study institution. Three-year all-cause mortality (i.e., mortality during follow-up up to 3 years after index admission) was identified where available. Utilization of critical care was identified, including both planned and unplanned admissions to level 2 or 3 care. The most recent preoperative serum ALB and lymphocyte count data were obtained from electronic patient records. The prognostic nutritional index (PNI) was calculated according to the formula described by Pinato et al. (serum albumin, g/L)  +  (0.005  ×  blood lymphocyte count, unit/µL) [3]. Patients were categorized as malnourished (PNI < 50) or not malnourished (PNI ≥ 50). This threshold was specified a priori to align with commonly used PNI stratification in the prior surgical literature and to facilitate comparability across studies; we did not re-optimize a cohort-specific cut-off in this dataset to avoid overfitting. The prognostic nutritional index (PNI) was calculated according to the original Onodera formulation: PNI = 10 × serum albumin (g/dL) + 0.005 × total lymphocyte count (/mm^3^). Because albumin in our laboratory system is reported in g/L (where 10 × albumin [g/dL] = albumin [g/L]) and lymphocytes are reported as cells/μL (numerically equivalent to /mm^3^), the calculation used in this study was equivalently expressed as follows: PNI = serum albumin (g/L) + 0.005 × total lymphocyte count (cells/μL). Patients were categorized as malnourished (PNI < 50) or not malnourished (PNI ≥ 50). The most recent preoperative serum albumin and lymphocyte count values were used to calculate the PNI; these were obtained on admission or within 24 h prior to surgery. All the data were collated and tabulated via Excel (Microsoft, Redmond, WA, USA).

### 2.5. Data Analysis

Comparative analyses were performed to evaluate the associations between nutritional status (malnourished vs. not malnourished) and surgical outcomes. Continuous variables were assessed for normality (Shapiro–Wilk test and visual inspection of histograms/Q–Q plots). As several key variables (including length of stay) were non-normally distributed, continuous data are presented as medians with interquartile ranges (IQRs) and compared using the Mann–Whitney U test. Because baseline characteristics differed between nutritional groups (including ASA grade, frailty score, and comorbidity burden), these variables were incorporated as covariates in the multivariable model to mitigate confounding when evaluating associations between PNI status and postoperative outcomes. Data were collated in Microsoft Excel. Inferential statistical analyses were performed using IBM SPSS Statistics (version 31, IBM Corp., Armonk, NY, USA). Normality was assessed using the Shapiro–Wilk test. Between-group comparisons were conducted using the Mann–Whitney U test for continuous variables and χ^2^ (or Fisher’s exact) tests for categorical variables. Multivariable modeling was performed to identify independent perioperative predictors of the major postoperative outcomes (stoma formation, prolonged hospital stay [≥12 days], 30-day readmission, and mortality [in-hospital or during follow-up]). All covariates were entered simultaneously (enter method) and retained irrespective of statistical significance: PNI group, age > 50 years, sex, ASA grade (3–5 vs. 1–2), frailty (CFS ≥ 5 vs. <5), comorbidity status (yes/no), sepsis (yes/no), tachycardia (HR ≥ 100 vs. <100), hypotension (SBP < 100 vs. ≥100), and pre- and postoperative NELA mortality risk (≥10% vs. <10%). Effect estimates are presented with 95% confidence intervals). Missing data were handled using a complete-case approach. Multivariate analysis of variance (MANOVA) was performed to identify independent perioperative predictors of the four major postoperative outcomes: stoma formation, prolonged hospital stay (≥12 days), 30-day readmission, and mortality (in-hospital or during follow-up). Prolonged LOS was defined as ≥12 days because 12 days represented the median LOS in the overall cohort, providing a pragmatic, data-driven threshold for categorical modeling.

Missing data were handled using a complete-case approach. Because the PNI cannot be derived when albumin and/or lymphocyte values are missing, these cases were excluded from analyses involving PNI. For multivariable models, cases with missing covariate and/or outcome data were excluded from the respective model (complete-case per model). No imputation was performed given the retrospective, audit-derived dataset and uncertainty regarding the missingness mechanism. Statistical significance was defined as *p* < 0.05. Kaplan–Meier survival analysis was used to assess three-year all-cause mortality, with differences between nutritional groups compared via the log-rank test. Denominators reflect complete-case availability for each outcome/model; where data were missing, analyses were performed on available complete cases (n reported alongside each analysis).

## 3. Results

### 3.1. Patient Demographics and Baseline Characteristics

A total of 482 patients who underwent emergency laparotomy between January 2019 and December 2023 were included. The cohort comprised 57% males, with a median age of 68 years (IQR 54–76). On the basis of the prognostic nutritional index (PNI), 318 patients (66%) were classified as malnourished (PNI < 50), and 164 (34%) were classified as not malnourished (PNI ≥ 50). The baseline characteristics were comparable in terms of age and sex distribution (*p* = 0.489 and 0.110, respectively). However, malnourished patients had significantly higher ASA grades (3–5) (*p* < 0.001), greater frailty (≥5) (*p* = 0.028), and a greater comorbidity burden (70% vs. 30%, *p* < 0.001) (Table 1).

### 3.2. Perioperative Characteristics and Patient Outcomes

Preoperative factors revealed that malnourished patients were more likely to present with hypotension (*p* = 0.010), although no significant associations were observed for tachycardia, sepsis, or predicted NELA mortality (all *p* > 0.05) (Table 2). Surgical interventions, including colorectal resection, small bowel resection, and adhesiolysis, were similarly distributed across groups (*p* = 0.873). However, malnourished patients were significantly more likely to require stoma formation (81% vs. 19%, *p* = 0.002) (Table 3).

Postoperative outcomes revealed a significantly longer hospital stay among malnourished patients. The median length of stay for patients with a PNI < 50 was 14 days (IQR 9–21), whereas it was 8 days (IQR 5–13) for those with a PNI ≥ 50; this difference was statistically significant (Mann–Whitney U = 17,892.0, *p* < 0.001). Overall, 75% of the malnourished patients experienced prolonged hospitalization (≥12 days), whereas 25% of the non-malnourished patients experienced prolonged hospitalization (*p* < 0.001).

While the incidence of postoperative complications was not significantly different between the groups (*p* = 0.992), malnourished patients had higher rates of 30-day readmission (88% vs. 13%, *p* = 0.026) and three-year all-cause mortality (74% vs. 26%, *p* = 0.044) (Table 4).

### 3.3. Multivariate Analysis

In multivariable modeling with simultaneous adjustment for PNI group, age >50 years, sex, ASA grade, frailty, comorbidity status, sepsis, tachycardia, hypotension, and pre- and postoperative NELA mortality risk, PNI ≥ 50 (vs. <50) was associated with lower odds of stoma formation and lower odds of prolonged length of stay (≥12 days) (Table 5). Associations between PNI and 30-day readmission and mortality during follow-up were not statistically significant after adjustment. Among covariates, sepsis was associated with stoma formation, higher ASA grade (3–5) was associated with prolonged LOS, and male sex was associated with mortality during follow-up (Table 5).

These results suggest that nutritional impairment is associated with frailty, physiological instability, and operative stress, leading to poorer recovery and an increased risk of death following emergency laparotomy (Table 5).

All covariates were entered simultaneously (PNI group, age > 50 years, sex, ASA grade, frailty category, comorbidity status, sepsis, tachycardia, hypotension, and pre- and postoperative NELA mortality risk); Table 5 reports effect estimates with 95% CIs for all predictors (including non-significant predictors).

Model covariates included PNI classification, ASA grade, frailty, comorbidities, hypotension, and sepsis (all entered simultaneously); Table 5 lists variables reaching statistical significance for each outcome. Effect magnitude is presented using partial η^2^ alongside test statistics.

The multivariable covariate set was prespecified and entered simultaneously for each outcome: PNI group, ASA grade, frailty (≥5), comorbidity status, hypotension, and sepsis. The “Significant predictors” column lists covariates reaching statistical significance for each outcome; non-significant covariates from the prespecified set are not repeated in the body of the table. Analyses were performed using complete cases for each outcome/model (denominators vary where data were missing). Observed power values are reported only as an SPSS output and are not interpreted as confirmatory evidence.

### 3.4. Survival Analysis

**Figure 1:**
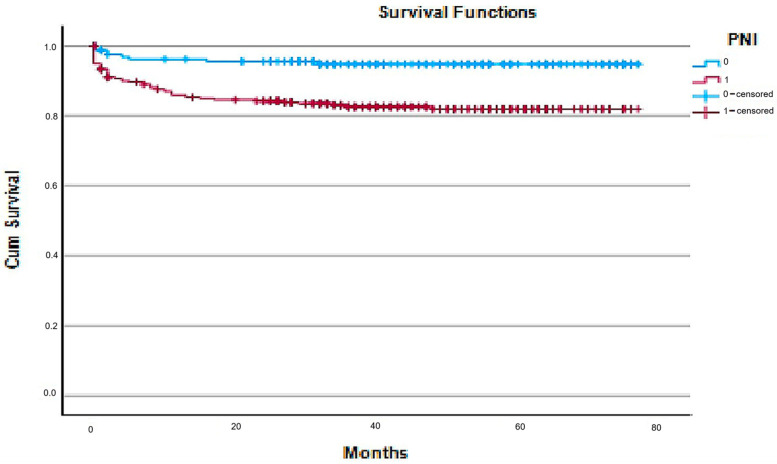
Kaplan–Meier survival curves showing reduced cumulative survival in patients with PNI < 50 compared with those with PNI ≥ 50. Kaplan–Meier survival curves comparing cumulative survival between malnourished (PNI < 50) and non-malnourished (PNI ≥ 50) patients following emergency laparotomy. The blue curve represents patients with a normal PNI, whereas the red curve represents malnourished patients. Censored observations are marked with “+”. A statistically significant difference in survival was observed between the groups (log-rank χ^2^ = 13.55, *p* < 0.01).

The median follow-up was 43 months (IQR 28–60). Survival estimates (including 3-year survival) were derived using Kaplan–Meier methods with right-censoring for patients who had not reached 3 years of follow-up (particularly those recruited later in the study period). During this period, 61 deaths (12.7%) occurred. Mortality was greater in the malnourished group (74% vs. 26%; χ^2^ = 4.01; *p* = 0.04). Kaplan–Meier survival analysis confirmed that patients with a PNI < 50 had significantly lower cumulative survival than those with a PNI ≥ 50 (log-rank χ^2^ = 13.55, *p* < 0.01) (Figure 1).

## 4. Discussion

This study demonstrated that malnutrition, as assessed by the prognostic nutritional index (PNI), was highly prevalent in patients undergoing emergency laparotomy and was significantly associated with adverse short- and long-term clinical outcomes. Notably, malnourished patients experienced longer hospital stays and were more likely to require stoma formation. Although unadjusted analyses indicated higher 30-day readmission rates and 3-year all-cause mortality rates in the malnourished cohort, these associations did not persist after multivariate adjustment, suggesting that malnutrition is a key but not isolated determinant of poor outcomes in this high-risk population.

This study underscores the critical prognostic relevance of the prognostic nutritional index (PNI) in patients undergoing emergency laparotomy, revealing a strikingly high prevalence of malnutrition (66%) within this cohort. Malnourished patients, as defined by a PNI < 50, presented a markedly elevated burden of physiological compromise, as evidenced by significantly higher ASA and frailty scores, increased comorbidities, and more frequent hypotensive presentations. These factors collectively reflect the intricate interplay between nutritional deficiency and systemic vulnerability in the emergency surgical setting.

Our findings align with previous evidence underscoring the predictive value of the The PNI combines serum albumin concentration and lymphocyte count to provide a reliable surrogate of both nutritional and immune status [1]. Numerous studies have validated its prognostic utility in elective colorectal, gastric, and hepatobiliary surgeries, where a lower PNI is correlated with increased postoperative morbidity and mortality [2,3]. However, few studies have examined its role in emergency surgical settings, which are typified by acute physiological derangement and lack preoperative optimization. Lee et al. reported a similar association between a low PNI and an increased risk of postoperative complications in emergency abdominal surgery [4], reinforcing the findings of the present study.

The current study builds on this limited body of work by evaluating a large, unselected cohort of emergency laparotomy patients over five years. The significant association between a low PNI and prolonged hospitalization observed here likely reflects both the physiological burden of malnutrition and the complexity of managing surgical complications in nutritionally depleted individuals. Moreover, the strong association between malnutrition and increased stoma formation may suggest a more cautious surgical approach in high-risk patients or reflect intraoperative findings such as bowel ischemia or contamination, which are more common in compromised hosts.

From a mechanistic perspective, malnutrition impairs collagen synthesis, delays wound healing, compromises immune defense, and reduces physiological reserves—factors critical in the postoperative period [6]. Emergency surgery patients often present in catabolic states with underlying sepsis or organ dysfunction, making even marginal nutritional deficits clinically significant. Despite these risks, current emergency surgical pathways often overlook routine nutritional screening, a gap highlighted by our data.

Interestingly, while malnourished patients presented increased unadjusted mortality and readmission rates, these outcomes were not independently associated with the PNI after controlling for confounders. This finding is consistent with studies demonstrating that, while the PNI is a valuable marker of perioperative vulnerability, it must be interpreted alongside clinical indicators such as the ASA score, frailty, and comorbid burden [5]. Notably, frailty and ASA score were also significantly associated with malnutrition in our cohort, underscoring the interconnected nature of these risk factors.

A key practical consideration is how nutritional risk is identified and managed as part of routine care in emergency laparotomy pathways. In elective surgery, structured pathways often incorporate formal nutritional screening and prehabilitation; however, in emergency settings, time constraints, acute physiological derangement, and limited collateral history can mean that malnutrition is under-recognized or addressed later in the admission. Where nutritional risk is identified, management typically centers on early dietetic involvement and timely nutrition support (preferably enteral when feasible), aligned with perioperative nutrition recommendations. In this context, a rapidly available blood-based index such as the PNI may function as a pragmatic trigger to prioritize nutritional review and escalation of support in the highest-risk patients, thereby improving the likelihood that nutritional vulnerability translates into actionable care.

These findings have important clinical and policy implications. First, they suggest that simple preoperative metrics such as the PNI should be incorporated into early risk stratification tools such as the NELA calculator, which currently emphasizes physiological and operative factors but lacks nutritional parameters [8]. Second, the results argue for the incorporation of prompt nutritional interventions—such as early enteral feeding or targeted immunonutrition—into perioperative pathways, even in acute surgical settings. Several randomized studies on elective surgery have shown that such interventions reduce infectious complications and shorten the length of stay [9,10].

Finally, this study contributes to the growing consensus that malnutrition is not merely a consequence of chronic illness but also a modifiable risk factor in surgical care. As emphasized in recent enhanced recovery after surgery (ERAS) guidelines, routine nutritional assessment and optimization should be standard even in emergent settings where feasible [11]. Malnutrition not only affects physiological resilience but also contributes to increased length of stay, postoperative complications, and healthcare costs [24]. Hospital malnutrition remains a pervasive issue globally, emphasizing the need for systematic nutritional screening and intervention in all surgical pathways [25].

Limitations include the retrospective, single-center design, which may limit generalizability. Residual confounding is possible because nutritional impairment is closely intertwined with frailty and comorbidity; although we adjusted for key measured covariates (ASA grade, frailty score, comorbidities, hypotension and sepsis), unmeasured factors may still influence outcomes. Given multiple outcomes, findings should be interpreted as hypothesis-generating, and we did not apply formal multiplicity correction. We did not include a contemporaneous elective laparotomy comparator cohort, and we could not undertake head-to-head comparisons between PNI and other screening tools (e.g., MUST/NRI/GNRI/CONUT) because required variables were not consistently available in the retrospective extract; prospective studies should collect these data to enable direct comparisons within emergency cohorts and to assess generalizability against elective populations. While PNI is simple and reproducible, it does not capture all dimensions of malnutrition (e.g., micronutrient status, sarcopenia and dietary intake) and can be influenced by acute inflammation, infection and resuscitation in emergency presentations. Published PNI thresholds vary by population and endpoint, and no universal cut-off is established; we therefore used a prespecified threshold for comparability, and future work should validate PNI-based risk stratification against accepted malnutrition standards (e.g., GLIM/SGA) and evaluate threshold performance in external cohorts. Postoperative nutritional management was not captured and could not be adjusted for, so treatment-related confounding is possible. Although we used a consistent preoperative sampling window (admission/within 24 h), the interval between sampling and skin incision was not consistently available, so timing-based sensitivity analyses were not undertaken. Associations with stoma formation should be interpreted in the context of intraoperative findings, disease severity and surgeon decision-making rather than as a direct causal effect of nutritional status. Exclusion of cases with missing albumin/lymphocyte measurements may introduce selection bias, and operative indication/diagnosis categories were heterogeneous and not uniformly coded at a granularity suitable for robust stratified analyses. Prospective, multicenter studies incorporating comprehensive nutritional assessment are needed to validate and extend these findings.

## 5. Conclusions

This study establishes the prognostic nutritional index (PNI) as a powerful predictor of outcome in patients undergoing emergency laparotomy, revealing that malnutrition—present in nearly two-thirds of this cohort—is both common and clinically consequential. Patients with a low PNI (<50) presented greater physiological compromise, reflected by higher ASA and frailty scores, more frequent hypotensive presentations, and increased comorbidity burden. These findings highlight the intrinsic link between poor nutritional status and reduced physiological resilience in acute surgical illness. Although operative strategies are similar across nutritional groups, malnourished patients face significantly longer hospital stays and are more likely to require stoma formation, underscoring the tangible impact of malnutrition on recovery and surgical complexity. While increased readmission and mortality rates did not remain statistically significant after adjustment, the PNI nevertheless emerged as a sensitive marker of overall clinical deterioration rather than an independent predictor of poor outcome.

Importantly, these results demonstrate that a simple, readily available blood-based metric can meaningfully inform perioperative risk assessment in emergency surgery, a field traditionally limited by the inability to optimize patients preoperatively. Integrating the PNI into existing frameworks, such as the NELA calculator, could enhance early decision-making, guide intraoperative caution, and prompt timely nutritional interventions. In an era emphasizing precision medicine and value-based care, the identification of malnutrition as a modifiable determinant of surgical risk is both a clinical and ethical priority. Future prospective studies should assess whether PNI-guided perioperative nutrition strategies can reduce complications, shorten hospitalization, and improve survival. Until such evidence is available, routine incorporation of PNI assessment into emergency laparotomy pathways offers a low-cost, high-yield opportunity to recognize nutritional vulnerability early and tailor management accordingly. By doing so, we can shift the paradigm of emergency surgical care from reactive to proactive, addressing malnutrition not as an inevitable consequence of illness but as a critical, modifiable target for improving outcomes.

## Figures and Tables

**Table 1:** Baseline patient characteristics according to nutritional status (PNI).

		Not Malnourished (PNI ≥ 50) n (%)	Malnourished (PNI < 50) n (%)	Chi-Square	*p* Value
Age	≤30	9 (39%)	14 (61%)	2.4233	0.489
	>30–50	22 (38%)	36 (62%)		
	>50–70	59 (37%)	101 (63%)		
	≥70	74 (31%)	167 (69%)		
Gender	Male	85 (31%)	189 (69%)	2.5507	0.110
	Female	79 (38%)	129 (62%)		
ASA	1–2	75 (44%)	96 (56%)	36.500	<0.001
	3–5	89 (29%)	222 (71%)		
Frailty Score	<5	142 (36%)	249 (64%)	4.8476	0.028
	≥5	22 (24%)	69 (76%)		
Comorbidities	Yes	117 (30%)	274 (70%)	15.520	<0.001
	No	47 (52%)	44 (48%)		

Baseline characteristics were compared between malnourished and non-malnourished patients via the chi-square test. ASA = American Society of Anaesthesiologists physical status classification. PNI = prognostic nutritional index.

**Table 2:** Perioperative characteristics according to nutritional status (PNI).

		Not Malnourished (≥50) n (%)	Malnourished (<50) n (%)	Chi-Square	*p* Value
**Preoperative NELA Mortality**	<10%	125 (36%)	219 (64%)	2.618	0.091
	≥10%	39 (28%)	99 (72%)		
**Preoperative Tachycardia**	<100	125 (35%)	234 (65%)	0.3951	0.530
	≥100	39 (32%)	84 (68%)		
**Preoperative Hypotension**	<100	6 (15%)	33 (85%)	6.568	0.010
	≥100	158 (36%)	285 (64%)		
**Sepsis (Preoperative)**	Yes	40 (29%)	97 (71%)	1.9873	0.159
	No	124 (36%)	221 (64%)		
**Postoperative NELA Mortality**	<10%	126 (36%)	225 (64%)	2.0173	0.156
	≥10%	38 (29%)	93 (71%)		

Perioperative variables according to PNI classification. NELA = National Emergency Laparotomy Audit-predicted mortality risk. *p* < 0.05 was considered significant.

**Table 3:** Surgical procedure details and nutritional status (PNI).

		Not Malnourished (≥50) n (%)	Malnourished (<50) n (%)	Chi-Square	*p* Value
**Procedure**	Colorectal resections (incl. Hartmann’s)	76 (34%)	150 (66%)	1.231	0.873
	Small bowel resection	13 (28%)	34 (72%)		
	Adhesiolysis	16 (35%)	30 (65%)		
	Exploratory or relook laparotomy	25 (36%)	45 (64%)		
	Other	34 (37%)	59 (63%)		
**Stoma Formation**	Yes	31 (19%)	130 (81%)	9.472	0.002 *
	No	66 (34%)	129 (66%)		
	Not applicable	67 (53%)	59 (47%)		

Distribution of surgical procedures and stoma formation according to nutritional status. * *p* < 0.05 was considered significant.

**Table 4:** Postoperative outcomes according to nutritional status (PNI).

		Not Malnourished (≥50) n (%)	Malnourished (<50) n (%)	Chi-Square	*p* Value
Length of Stay (LOS)	≥12 days	63 (25%)	193 (75%)	21.5621	<0.001
	<12 days	101 (45%)	125 (55%)		
All Complications	Yes	47 (34%)	91 (66%)	0.0001	0.992
	No	117 (34%)	227 (66%)		
Planned Level 2/3 Admission	Yes	88 (32%)	183 (68%)	0.6648	0.415
	No	76 (36%)	135 (64%)		
Unplanned Level 2/3 Admission	Yes	49 (37%)	85 (63%)	0.5344	0.465
	No	115 (33%)	233 (67%)		
30-Day Readmission	Yes	3 (13%)	21 (88%)	Fisher’s	0.026
	No	161 (35%)	297 (65%)		
3-Year All-Cause Mortality	Yes	28 (26%)	80 (74%)	4.067	0.044
	No	136 (36%)	238 (64%)		
Mortality During Admission	Yes	11 (22%)	38 (78%)	3.256	0.712
	No	153 (35%)	280 (65%)		

Postoperative outcomes comparing malnourished vs. non-malnourished patients on the basis of the PNI. LOS = length of stay. Statistical significance was determined via the chi-square test or Fisher’s exact test as appropriate.

**Table 5:** Multivariable model parameter estimates for major postoperative outcomes. Unstandardized coefficients (B) with 95% confidence intervals are shown for all covariates entered simultaneously. Reference category for PNI is PNI < 50. Analyses were performed using complete cases for the multivariable model.

Predictor	B (95% CI)	*p*	Partial η^2^
**(A) Stoma formation**			
PNI ≥ 50 (vs. PNI < 50)	−0.180 (−0.300 to −0.059)	0.004	0.025
ASA 3–5 (vs. ASA 1–2)	−0.015 (−0.144 to 0.115)	0.825	0.000
Frailty ≥ 5 (vs. <5)	−0.039 (−0.188 to 0.109)	0.604	0.001
Comorbidity present (vs. none)	−0.064 (−0.211 to 0.084)	0.398	0.002
Hypotension (SBP < 100 mmHg)	−0.129 (−0.345 to 0.086)	0.238	0.004
Sepsis present	0.191 (0.061 to 0.321)	0.004	0.024
Age > 50 years	−0.008 (−0.167 to 0.151)	0.918	0.000
Male sex	0.011 (−0.096 to 0.117)	0.844	0.000
Tachycardia (HR ≥ 100)	−0.031 (−0.166 to 0.103)	0.647	0.001
Pre-op NELA mortality ≥ 10%	0.015 (−0.254 to 0.285)	0.912	0.000
Post-op NELA mortality ≥ 10%	0.083 (−0.190 to 0.356)	0.549	0.001
**(B) Prolonged LOS (≥12 days)**			
PNI ≥ 50 (vs. PNI < 50)	−0.226 (−0.343 to −0.109)	<0.001	0.041
ASA 3–5 (vs. ASA 1–2)	0.211 (0.086 to 0.337)	<0.001	0.032
Frailty ≥ 5 (vs. <5)	−0.023 (−0.167 to 0.120)	0.748	0.000
Comorbidity present (vs. none)	−0.070 (−0.213 to 0.073)	0.336	0.003
Hypotension (SBP < 100 mmHg)	0.120 (−0.088 to 0.329)	0.257	0.004
Sepsis present	−0.005 (−0.131 to 0.121)	0.941	0.000
Age > 50 years	0.127 (−0.027 to 0.281)	0.105	0.008
Male sex	−0.048 (−0.151 to 0.055)	0.363	0.002
Tachycardia (HR ≥ 100)	−0.012 (−0.142 to 0.118)	0.857	0.000
Pre-op NELA mortality ≥ 10%	0.078 (−0.183 to 0.339)	0.558	0.001
Post-op NELA mortality ≥ 10%	−0.085 (−0.349 to 0.179)	0.527	0.001
**(C) 30-day readmission**			
PNI ≥ 50 (vs. PNI < 50)	−0.021 (−0.072 to 0.029)	0.403	0.002
ASA 3–5 (vs. ASA 1–2)	0.004 (−0.050 to 0.058)	0.882	0.000
Frailty ≥ 5 (vs. <5)	0.040 (−0.021 to 0.102)	0.198	0.005
Comorbidity present (vs. none)	0.040 (−0.022 to 0.101)	0.203	0.005
Hypotension (SBP < 100 mmHg)	−0.019 (−0.109 to 0.070)	0.670	0.001
Sepsis present	0.007 (−0.047 to 0.061)	0.803	0.000
Age > 50 years	0.013 (−0.053 to 0.079)	0.707	0.000
Male sex	−0.024 (−0.069 to 0.020)	0.279	0.003
Tachycardia (HR ≥ 100)	−0.031 (−0.087 to 0.025)	0.271	0.004
Pre-op NELA mortality ≥ 10%	−0.034 (−0.146 to 0.077)	0.545	0.001
Post-op NELA mortality ≥ 10%	0.026 (−0.087 to 0.140)	0.647	0.001
**(D) Mortality during follow-up**			
PNI ≥ 50 (vs. PNI < 50)	−0.049 (−0.144 to 0.045)	0.308	0.003
ASA 3–5 (vs. ASA 1–2)	0.076 (−0.025 to 0.178)	0.139	0.006
Frailty ≥ 5 (vs. <5)	0.035 (−0.081 to 0.152)	0.550	0.001
Comorbidity present (vs. none)	0.009 (−0.106 to 0.125)	0.874	0.000
Hypotension (SBP < 100 mmHg)	0.042 (−0.127 to 0.210)	0.626	0.001
Sepsis present	−0.023 (−0.125 to 0.078)	0.650	0.001
Age > 50 years	0.016 (−0.108 to 0.141)	0.795	0.000
Male sex	0.096 (0.012 to 0.179)	0.025	0.015
Tachycardia (HR ≥ 100)	0.031 (−0.075 to 0.136)	0.566	0.001
Pre-op NELA mortality ≥ 10%	0.203 (−0.008 to 0.414)	0.060	0.010
Post-op NELA mortality ≥ 10%	0.051 (−0.163 to 0.265)	0.641	0.001

## Data Availability

All relevant data and results included in this article have been published along with the article.
